# Impact of Anti—Vascular Endothelial Growth Factor Treatment on Neovascular Age-Related Macular Degeneration with and without Retinal Pigment Epithelial Detachment: A Real-World Study

**DOI:** 10.3390/jpm14101041

**Published:** 2024-09-28

**Authors:** Yu-Wei Kuo, Cheng-Yung Lee, Yi-Ting Hsieh, Chung-May Yang, Tzyy-Chang Ho, Tso-Ting Lai, Chang-Hao Yang

**Affiliations:** 1 Department of Ophthalmology, National Taiwan University Hospital, Taipei City 100225, Taiwan; cgh17847@cgh.org.tw (Y.-W.K.); ythyth@ntu.edu.tw (Y.-T.H.);; 2 Department of Ophthalmology, Sijhih Cathay General Hospital, New Taipei City 221037, Taiwan; 3 Department of Ophthalmology, Cathay General Hospital, Taipei City 106438, Taiwan; 4 School of Public Health, Taipei Medical University, Taipei City 110301, Taiwan; 5 Department of Ophthalmology, National Taiwan University Hospital Hsin-Chu Hospital, Hsinchu City 300195, Taiwan; 6 Graduate Institute of Clinical Medicine, College of Medicine, National Taiwan University, Taipei City 100233, Taiwan; 7 Department of Ophthalmology, College of Medicine, National Taiwan University, Taipei City 100233, Taiwan

**Keywords:** anti-vascular endothelial growth factor, neovascular age-related macular degeneration, retinal pigment epithelial detachment, optical coherence tomography, best-corrected visual acuity

## Abstract

Background/Objectives: This study evaluates the impact of anti-vascular endothelial growth factor (anti-VEGF) treatment on neovascular age-related macular degeneration (nAMD) with and without pigment epithelial detachment (PED) over a one-year period. Methods: Conducted at a tertiary referral center in Taiwan, this retrospective analysis included 88 eyes treated with intravitreal aflibercept injections. Patients were categorized into four groups based on the presence or absence of PED at baseline and 12 months post-treatment. Results: Significant reductions in central macular thickness (CMT) and PED height were observed, although no statistical difference was found in best-corrected visual acuity (BCVA). The presence or type of PED did not negatively impact visual outcomes. Among nAMD patients with persistent PED throughout the first year of anti-VEGF treatment, linear regression analysis showed that mixed-type PED revealed poor final BCVA compared to those with serous PED. The analysis also identified older age and poorer initial BCVA as predictors of less favorable visual outcomes. Conclusions: This study highlights the effectiveness of anti-VEGF therapy in real-world settings and offers insights into factors influencing visual outcomes for nAMD patients with PED.

## 1. Introduction

Age-related macular degeneration (AMD) is a leading cause of vision loss among the elderly, particularly in developed countries [1]. In 2020, the global population affected by AMD was estimated at 196 million, with this number projected to reach 288 million by 2040 [2]. As life expectancy increases and the global population ages, the incidence of AMD is anticipated to grow, posing a major public health challenge. Other associated risk factors include female sex, genetic factors, Caucasian race, obesity, smoking, alcohol consumption, and dietary habits [3,4,5,6].

Neovascular AMD (nAMD), also known as wet or exudative AMD, accounts for 90% of vision loss associated with AMD. The neovascular form of AMD presents unique challenges for clinicians, often coexists with pigment epithelial detachment (PED), and complicates treatment options [7]. Among patients with nAMD, the prevalence of PED accounts for 30% to 80% [8,9,10,11,12,13,14,15]. PED refers to the anatomical separation of the retinal pigment epithelium (RPE) from the Bruch’s membrane [16,17]. Within the sub-RPE space, there may be serous exudate, hemorrhagic or fibrovascular tissue, or drusenoid material, identified according to their clinical appearance, optical coherence tomography (OCT), and fluorescein angiography (FA) [18,19]. With OCT, serous PED is characterized by a smooth, domed elevation of the RPE with a sharp angle of pigment epithelial layer detachment over an optically empty space, with Bruch’s membrane visible below [20]. In contrast, hemorrhagic PEDs resemble serous PEDs in appearance but are distinguished by their dark gray or black color, which indicates the presence of blood [21]. Fibrovascular PEDs are identified by RPE elevation with non-homogeneous, mild to moderate hyper-reflectivity, while drusenoid PEDs are characterized by an undulating, hyper-reflective RPE band over a moderately hyper-reflective mass corresponding to confluent drusen [22].

Regardless of the PED type, all PEDs generally follow a similar clinical course, with approximately 50% of patients experiencing visual loss within one year of follow-up if left untreated [23]. Therefore, the presence of PED is considered a significant predictor of vision loss in AMD [12,14]. Some researchers suggest that the baseline volume of PED could serve as a predictor for both short-term and long-term visual outcomes [24]. However, the effect of PED appearance and its specific types on these outcomes remains debated.

The earliest treatments for nAMD were argon laser photocoagulation and photodynamic therapy, which have now been largely replaced by anti-vascular endothelial growth factor (anti-VEGF) therapy [25,26]. In recent decades, intravitreal injection of anti-VEGF has become the first-line treatment for nAMD [27,28,29,30]. Currently, several anti-VEGF drugs have been proven effective in clinical trials. These include brolucizumab (Beovu), ranibizumab (Lucentis), aflibercept (Eylea), and the off-label use of bevacizumab (Avastin) [31,32]. Consistent treatment with anti-VEGF agents leads to improved visual acuity, reduced macular thickness, slowed disease progression, and also stabilized anatomical parameters [7,33]. However, while intravitreal anti-VEGF treatment can reduce or eliminate intraretinal and subretinal fluid, PEDs may exist persistently [34].

Despite recent advances, there remains a significant gap in the literature concerning the long-term visual outcomes of nAMD patients with PED undergoing anti-VEGF treatments. This is especially concerning given the rising prevalence of nAMD and the frequent occurrence of PED [35,36]. The lack of comprehensive studies identifying predictive factors for long-term visual outcomes leaves clinicians with limited guidance for personalized treatment planning. Moreover, current research has yet to address how treatment outcomes may vary based on different types of PED.

This study aims to fill these gaps by conducting a retrospective analysis of nAMD patients treated with aflibercept injections at a tertiary university hospital. Patients will be categorized based on the presence or absence of PED. The characteristics of PED in treatment-naïve nAMD patients over a 12-month period following initial aflibercept treatment will be evaluated after a one-year follow-up.

## 2. Materials and Methods

### 2.1. Study Design

This retrospective study conducted in a tertiary referral center in Taiwan aims to evaluate the efficacy of aflibercept in naïve nAMD with and without PED. The secondary objective was to identify predictive factors influencing visual outcomes and PED diameters after one year.

### 2.2. Study Population

Patients who visited the retina clinic at National Taiwan University Hospital between September 2019 and October 2021, were treatment-naïve for nAMD, and received subsequent standard anti-VEGF treatment were included. These patients had no prior history of receiving any treatment for nAMD and were carefully monitored throughout the course of their anti-VEGF therapy to assess treatment outcomes and disease progression. To avoid bias from differences in therapeutic efficacy of anti-VEGF medications, only patients treated with aflibercept were included, while those who received ranibizumab or bevacizumab were excluded. All patients received insurance reimbursement for intravitreal aflibercept injections from the National Health Insurance (NHI) of Taiwan. The NHI criteria include: (1) being 50 years of age or older, (2) baseline best-corrected visual acuity (BCVA) between 0.05 and 0.5 (decimal, equal to 20/40 to 20/400), (3) active nAMD lesions confirmed by both fluorescein angiography and OCT without evidence of other causes of neovascularization such as polypoidal choroidal vasculopathy, uveitis, myopic choroidal neovascularization, or other secondary choroidal neovascularization, (4) no evidence of geographic atrophy or macular scar on either OCT or fluorescence angiography. These criteria ensured a consistent patient profile for the study, minimizing variability in treatment eligibility. In addition to NHI criteria, a minimum follow-up time of 12 months was required for enrollment in our study. Patients were also excluded if they had received other treatments for nAMD (including other anti-VEGF agents, steroids, or photodynamic therapy) prior to the first injection. Other exclusion criteria included epiretinal membrane, diabetic macular edema, and central or branch retinal vein occlusion. These exclusions ensured the study focused solely on patients with a clear diagnosis of nAMD and sufficient follow-up time for reliable evaluation of treatment efficacy. The study adhered to the principles of the Declaration of Helsinki and was approved by the National Taiwan University Hospital Research Ethics Committee (IRB 202207141RIN). Written informed consent was waived due to the retrospective nature of the study.

### 2.3. Data Collection and Outcome Measurement

**Figure 1:**
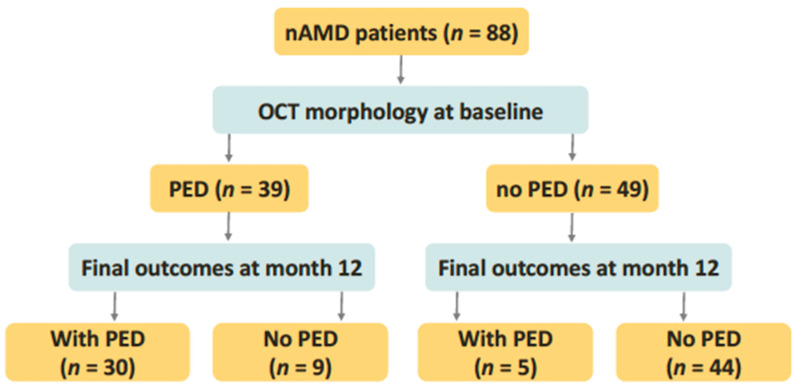
Flowchart of patient categorization. A total of 88 patients with neovascular age-related macular degeneration were included in the study, 39 of whom had pigment epithelial detachment (PED) as determined by optical coherence tomography images before aflibercept treatment. Patients were categorized into four groups based on the presence or absence of PED before treatment and at the 12-month follow-up. Abbreviations: nAMD, neovascular age-related macular degeneration; OCT, optical coherence tomography; PED, pigment epithelial detachment.

Patient demographic data including age and sex were collected from medical records. The BCVA measurements were recorded at baseline and at 12 months after the first injection and were converted to logMAR scores for calculations. OCT examinations were performed at baseline and at 12-month follow-up visit. Central macular thickness (CMT) was measured using the macular thickness map program integrated into the SD-OCT systems (Cirrus™ HD-OCT, Carl Zeiss Meditec, Inc., Dublin, CA, USA; or RTVue^®^ Model-RT100 version 3.5, Optovue, Inc., Fremont, CA, USA). PED height was manually assessed using a built-in caliper tool, measuring from the highest point of the RPE to an ideal RPE line. They were categorized into four groups based on the presence or absence of PED at baseline and at the 12-month follow-up: Group 1 (PED at both time points), Group 2 (PED at baseline but not at 12-month follow-up), Group 3 (no PED at baseline but PED at 12-month follow-up), and Group 4 (no PED at either time point) (Figure 1). PED types were classified as serous, hemorrhagic, drusenoid, or mixed-type.

### 2.4. Treatment Protocol

All patients received an initial series of three-monthly loading injections of aflibercept, followed by additional injections based on need. The criteria for “as needed” involved continuing monthly injections until fluid resolution. Treatment was then only resumed upon the appearance of recurrent exudation or PED appeared. The patients were monitored monthly from the first injection through the 12th month, with all assessments conducted by the same physicians after the loading dose.

### 2.5. Statistical Analysis

Data were presented as the mean with standard deviation (SD). Pre-treatment and post-treatment BCVA, CMT, and PED height were analyzed using paired sample *t*-tests. Subgroup analyses were conducted using the Kruskal–Wallis test and chi-squared test, while comparisons between two groups were performed using the Mann–Whitney U test and chi-squared test. Linear regression analysis was used to identify predictive factors for visual outcomes after one year. Statistical significance was defined as a *p*-value of less than 0.05. Data analysis was performed using SPSS Version 26.0.

## 3. Results

### 3.1. Study Population and Demographics

This study enrolled a total of 88 eyes from patients undergoing aflibercept treatment for nAMD. There were 30 eyes in Group 1, 9 in Group 2, 5 in Group 3, and 44 in Group 4 (Figure 1). Table 1 presents the demographics and treatment outcomes of the study participants. The mean age of participants was 73.2 years (SD = 9.8), with males comprising 44.3% of the cases. Notably, 44.3% (39 cases) exhibited pigment epithelial detachment (PED).

### 3.2. Treatment Responses at Month 12

After an average of five intravitreal injections of aflibercept over one year, there was no statistically significant difference (*p* = 0.563) between the mean baseline BCVA of 0.765 logMAR (SD = 0.463) and the mean BCVA at the 12th month of treatment, which was 0.792 logMAR (SD = 0.580) (Table 1). Conversely, a significant reduction in central macular thickness (CMT) was observed (*p* < 0.001) from a mean baseline CMT of 324.4 μm (SD = 112.5) to a CMT of 237.1 μm (SD = 72.1) at the 12th month of treatment.

Among the 39 patients with PED, the mean baseline PED height was 351.8 μm (SD = 259.6), which decreased to 212.6 μm (SD = 255.9) after one year of aflibercept treatment. This reduction in mean PED height was statistically significant (*p* = 0.006).

As demonstrated in Table 1, Group 1 demonstrated the least improvement in CMT, which was significantly lower than Group 4 (*p* = 0.023). Compared to the baseline CMT between Groups 1 and 4, Group 4 presented significantly thicker CMT and more pronounced CMT improvement at 12 months than Group 1 (*p* = 0.013, *p* = 0.003) (Table 1, last column).

### 3.3. Prediction of BCVA at Month 12 Based on PED Presence and Type

Table 2 depicts the various types of PED observed in Group 1 and Group 2, and evaluates the predictive value of PED type in PED resolution. Neither the presence nor the type of PED was found to have a detrimental impact on visual outcomes (*p* = 0.329). Although the number of patients in Group 2 was smaller; we infer that with appropriate treatment, favorable visual outcomes can be expected regardless of PED type.

### 3.4. Additional Predictive Factors for BCVA at Month 12

Linear regression analysis was conducted to assess potential predictive factors for final BCVA at the one-year mark in patients with persistent PED throughout the first year of aflibercept treatment (Group 1) (Table 3). The analysis revealed that older age and worse initial BCVA were associated with poorer visual outcomes (β = 0.500 and 2.712 respectively; *p* = 0.031 and 0.012, respectively). Additionally, mixed-type PED was also associated with worse final vision compared to those with serous PED (β = 1.102, *p* = 0.035). Conversely, sex, CMT, and PED height did not show a statistically significant correlation with final BCVA in the linear regression model.

Linear regression analysis with interaction terms showed that the presence of mixed-type PED reduces the correlation between baseline BCVA and final BCVA, with the beta value decreasing from 2.712 by 1.384 to 1.329 (Group 1) (Table 4). Furthermore, the existence of mixed-type PED negatively impacts visual prognosis (β = 1.102, *p* = 0.035) (Group 1) (Table 3).

## 4. Discussion

PED is present in 44.3% of nAMD cases in our study, which is compatible with previous reports [8,9,10,11,12,13,14,15]. The study provides compelling evidence for the efficacy of aflibercept treatments in naïve nAMD patients, both with and without PED. Two key results are noteworthy. There was a marked reduction in CMT and a considerable decrease in PED height over one year, and the presence or type of PED did not influence visual outcomes over one year. The findings align with the current understanding that anti-VEGF treatments are highly effective in reducing macular thickness for nAMD patients.

Since intravitreal injection of anti-VEGF has become the first-line treatment for nAMD, several anti-VEGF agents—such as brolucizumab (Beovu), ranibizumab (Lucentis), aflibercept (Eylea), and bevacizumab (Avastin)—have been widely used to treat these patients [31]. Despite ongoing debate about the effectiveness of various anti-VEGF agents for nAMD, some studies have specifically compared aflibercept and ranibizumab for treating PED. One study found that aflibercept appears to be superior to ranibizumab in improving BCVA, reducing PED height, and achieving regression of PED with fewer injections in patients with nAMD and PED [37]. Other research has reported that both aflibercept and ranibizumab resulted in a reduction of all PED dimensions; however, larger, higher, and more hypo-reflective PEDs showed better anatomical responses, particularly with aflibercept [38]. Additionally, patients with exudative age-related macular degeneration who were resistant to bevacizumab and ranibizumab demonstrated significant visual improvement when switched to aflibercept, along with a sustained reduction in central macular thickness over 12 months of follow-up [39]. Since aflibercept is the most commonly used anti-VEGF agent for treating nAMD patients in our hospital, this study focused on aflibercept to eliminate potential bias from the varying therapeutic efficacies of different anti-VEGF agents.

According to the previous literature reporting on the visual prognosis and treatment outcomes of anti-VEGF therapy for nAMD, William et al. provide evidence of the morphological improvements in the macula under anti-VEGF treatment, particularly highlighting significant gains in BCVA and foveal central thickness in treatment-naive eyes [33]. Similarly, Zhou et al. demonstrated the effectiveness of anti-VEGF therapy in significantly reducing central retinal thickness [40]. In our study, however, there was no statistically significant improvement in visual acuity after one year of treatment. This may be attributed to the PRN protocol, the limited number of injections in a real-world setting, and the relatively small sample size. Despite a lower number of injections in the first year of treatment possibly contributing to poorer visual improvement [41], the average of approximately five injections in this study’s first year is comparable to that reported in other real-world studies (also around five injections in the first year) [42,43].

To explore the impact of PED on visual outcomes, numerous studies have investigated the relationship between the presence or absence of PED and visual outcomes, yielding varied conclusions. There were studies that reported the presence of PED in nAMD patients does not affect BCVA [8,9,11,12,13,44,45,46,47,48]. The EXCITE [12] and MONT BLANC [11] studies also noted that PEDs represented a negative effect on baseline BCVA levels and during follow-up only when combined with intraretinal cysts and subretinal fluid. Post hoc analysis of EXCITE [13] also found no significant relationship between baseline PED presence and final visual outcome. Some other studies, however, reported better visual outcome when PED coexists with nAMD. Sarraf et al., in a post hoc analysis of the HARBOR study, reported that patients with baseline PED had better BCVA at both baseline (55.7 ETDRS letters) and 24 months (64.4 ETDRS letters) compared to those without baseline PED (51.9 and 62.0 ETDRS letters, respectively) [10]. Still other studies suggested negative impacts on BCVA outcomes in AMD patients when PED was present [14,15]. In the VIEW 1 [14] analysis, baseline PED was associated with a 1.88-letter loss in BCVA (ETDRS) by week 52. Similarly, in VIEW 2 [15], PED alone led to a 1.1-letter loss from weeks 52 to 96, with an additional 2.5- to 4.4-letter decrease when intraretinal cysts were present. Moreover, William et al. showed that PED was more frequently found in eyes that were refractory to VEGF treatment [33]. In our study, the presence of PED neither affected baseline BCVA nor BCVA after one year of treatment. On the other hand, our study indicated that the presence of PED contributed to less improvement in CMT after one year of treatment (Table 1, last column).

In studies examining predictive factors of PED morphology, Blanco-Garavito et. al. reported a negative correlation between PED height and BCVA [49], while others found no significant relationship between maximal height and BCVA [44,50,51,52,53,54,55,56]. Additionally, Cheong et al. reported on various morphological factors, such as height, width, volume, retinal pigment epithelium (RPE) tear, or cholesterol bands on OCT, which did not significantly impact visual outcomes at 12 months [57]. In addition, some studies further examined the contents of PED. While no significant differences in BCVA between serous and vascular PED were reported [55,58,59], others found that visual outcomes were best for serous PED compared to fibrovascular, hemorrhagic, and mixed-type [60]. In our study, among nAMD patients with persistent PED throughout the first year of aflibercept treatment, (Group 1), those with mixed-type PED showed worse vision compared to those with serous PED, while other types of PED did not exhibit statistical differences in terms of BCVA when compared to serous PED (Table 3). Furthermore, older age and worse baseline BCVA were significantly associated with poorer final visual acuity.

This study has several strengths and limitations. One key strength is the homogenous group of patients treated consistently with the same anti-VEGF agent, aflibercept. This is particularly noteworthy as the study demonstrated that aflibercept led to favorable anatomical outcomes in patients with PED. However, the limitations include a lower number of injections administered during the first year of treatment, a relatively short observation period, and a smaller sample size in some groups. These factors limit the ability to draw long-term conclusions about the progression of PED in nAMD over several years or decades. In addition, there are now newer anti-VEGF drugs like faricimab being used to treat nAMD patients. Recent studies have shown that the treatment effects of these two drugs on PED are comparable, with the maximum height of PED after each loading dose being similar [61]. In the future, we hope to include more patients with PED and various drug treatments to provide more information. Nevertheless, our study underscores the importance of characterizing PED in treatment-naïve nAMD patients. Given the high prevalence of PED in nAMD, our findings can offer valuable insights for the initial management of newly diagnosed nAMD patients with PED, particularly regarding expectations for long-term visual outcomes.

In summary, our study showed that intravitreal aflibercept injections effectively reduce CMT and PED height of nAMD. However, no significant improvement in BCVA was observed after one year of treatment. The presence of PED did not impact baseline BCVA or BCVA at 12 months in nAMD. Among nAMD patients with persistent PED throughout the first year of anti-VEGF treatment, mixed-type PED may lead to inferior final visual acuity compared to cases with serous PED. Older age and worse baseline BCVA may also contribute to poorer final vision in these patients.

## Figures and Tables

**Table 1:** Demographics and treatment outcomes across four groups with different characteristics of pigment epithelial detachment.

Group, No.	1	2	3	4	Total	*p* ^1,2^	*p* ^1,3^
Patient number, *n*	30	9	5	44	88	*-*	*-*
Age, years	75.8 (8.4)	72.7 (5.0)	70.6 (7.4)	71.9 (11.3)	73.2 (9.8)	0.359	0.149
Sex, male/female	12:18	6:3	1:4	20:24	39:49	0.352	0.642
Baseline BCVA before aflibercept treatment, logMAR	0.621 (0.337)	0.841 (0.501)	0.580 (0.383)	0.869 (0.517)	0.765 (0.463) ^4^	0.181	0.056
BCVA at month 12 of aflibercept treatment, logMAR	0.641 (0.521)	1.106 (0.632)	0.564 (0.579)	0.856 (0.589)	0.792 (0.580) ^4^	0.127	0.129
BCVA changes, logMAR	0.020 (0.464)	0.265 (0.373)	−0.016 (0.315)	−0.012 (0.429)	0.03 (0.436)	0.242	0.925
Baseline CMT before aflibercept treatment, μm	286.1 (93.6)	378.3 (149.1)	339.6 (120.8)	337.8 (111.1)	324.4 (112.5) ^5^	0.066	0.013 *
CMT at month 12 of aflibercept treatment, μm	256.7 (86.1)	263.8 (88.0)	194.8 (15.6)	224.2 (55.9)	237.1 (72.1) ^5^	0.217	0.121
CMT changes, μm	−29.4 (103.9)	−114.6 (158.7)	−144.8 (121.7)	−113.5 (113.6)	−88.4 (119.4)	0.023 *	0.003 *
Total injections, times	5.1 (2.1)	6.4 (1.7)	4.6 (3.4)	4.6 (2.0)	5.0 (2.1)	0.110	0.352

^1^ * *p* < 0.05. ^2^ The *p*-value in this column was derived from a Kruskal–Wallis test or chi-squared test comparing each variable across all groups. ^3^ The *p*-value in this column was derived from a Mann–Whitney U test or chi-squared test comparing each variable between Group 1 and Group 4. ^4^ A paired *t*-test comparing the mean baseline BCVA to BCVA at the 12th month of treatment showed no statistical significance (*p* = 0.563). ^5^ There was a statistically significant reduction in mean CMT at the 12th month of treatment compared to mean baseline CMT, as determined by a paired *t*-test (*p* < 0.001). All data, except for sex categories and patient numbers, are presented as means (SD). Abbreviations: BCVA = best-corrected visual acuity, CMT = central macular thickness.

**Table 2:** Characteristics of pigment epithelial detachment between Group 1 and Group 2.

Group, No.		1	2	Total	*p*
Patient number, *n*		30	9	39	-
Types of PED	Serous PED, *n*	11	3	14	0.329
	Hemorrhagic PED, *n*	6	4	10	
	Drusenoid PED, *n*	6	0	6	
	Mixed-type, *n*	7	2	9	

Abbreviations: PED = pigment epithelial detachment.

**Table 3:** Linear regression analysis for factors associated with final visual acuity in neovascular age-related macular degeneration with pigment epithelial detachment.

Independent Variables of the Linear Regression Model ^1^		β Coeffiecient	*p* ^2^	Confidence Interval	
				Lower	Upper
Age		0.500	0.031 *	0.003	0.059
Sex ^3^		0.117	0.500	−2.52	0.496
Baseline BCVA		2.712	0.012 *	1.073	7.307
Baseline CMT		0.491	0.381	−0.004	0.009
PED height		0.247	0.788	−0.004	0.005
PED type ^4^	Hemorrhagic	0.269	0.827	−2.945	3.634
	Drusenoid	0.295	0.604	−1.134	1.890
	Mixed-type	1.102	0.035 *	0.103	2.565

^1^ The adjusted R^2^ for the linear regression model is 0.395. ^2^ * *p* < 0.05. ^3^ A dichotomous variable was utilized, where males were assigned a value of 1 and females were assigned a value of 0. ^4^ In the regression model, all patients included had one of four types of PED. Three dummy variables were created and assigned to hemorrhagic PED, drusenoid PED, and mixed-type PED for comparison against serous PED. Abbreviations: BCVA = best-corrected visual acuity, CMT = central macular thickness, PED = pigment epithelial detachment.

**Table 4:** Linear regression analysis, including interaction terms for factors associated with final visual acuity in neovascular age-related macular degeneration with pigment epithelial detachment.

Independent Variables of the Linear Regression Model ^1,3−5^		β Coeffiecient	*p* ^2^	Confidence Interval	
				Lower	Upper
Interaction terms	Baseline BCVA × Baseline CMT	−1.691	0.102	−0.013	0.001
	Baseline BCVA × PED height	−0.395	0.710	−0.009	0.007
	Baseline BCVA × Hemorrhagic type PED	−0.990	0.480	−5.174	2.540
	Baseline BCVA × Drusenoid type PED	−1.223	0.103	−4.497	0.454
	Baseline BCVA × Mixed-type PED	−1.383	0.013 *	−5.296	−0.740

^1^ A multivariate linear regression model with interaction terms was constructed. The formula for the model was: $Y(Final\;Visual\;Acuity)=\beta_{0}+\beta_{1}\times Age+\beta_{2}\times Sex+\beta_{3}\times Baseline\;BCVA+\beta_{4}\times Baseline\;CMT+\beta_{5}\times PED\;height+\beta_{6}\times Hemorragic\;PED+\beta_{7}\times Drusenoid\;PED+\beta_{8}\times Mixed-type\;PED+\beta_{9}\times \left(Baseline\;BCVA\times Baseline\;CMT\right)+\beta_{10}\times \left(Baseline\;BCVA\times PED\;height\right)+\beta_{11}\times \left(Baseline\;BCVA\times Hemorrhagic\;type\;PED\right)+\beta_{12}\times \left(Baseline\;BCVA\times Mixed-type\;PED\right)+\varepsilon$. Here, Y denotes final visual acuity. Variables with coefficients $\beta_{5}$ to $\beta_{7}$ represent dummy variables for four PED types (see ^5^), and those followed coefficient $\beta_{9}$ to $\beta_{12}$ represent interaction terms involving baseline BCVA. ε accounts for error. ^2^ * *p* < 0.05. ^3^ The adjusted R^2^ for the linear regression model is 0.395. ^4^ A dichotomous variable was utilized, where males were assigned a value of 1 and females were assigned a value of 0. ^5^ In the regression model, all patients included had one of four types of PED. Three dummy variables were created and assigned to hemorrhagic PED, drusenoid PED, and mixed-type PED for comparison against serous PED. Abbreviations: BCVA = best-corrected visual acuity, CMT = central macular thickness, PED = pigment epithelial detachment.

## Data Availability

Data are contained within the article.

## References

[1] Arslan J., Benke K.K. (2022). Application of Machine Learning to Ranking Predictors of Anti-VEGF Response. Life.

[2] Wong W.L., Su X., Li X., Cheung C.M., Klein R., Cheng C.Y., Wong T.Y. (2014). Global prevalence of age-related macular degeneration and disease burden projection for 2020 and 2040: A systematic review and meta-analysis. Lancet Glob. Health.

[3] Hyman L., Neborsky R. (2002). Risk factors for age-related macular degeneration: An update. Curr. Opin. Ophthalmol..

[4] Ristau T., Ersoy L., Hahn M., den Hollander A.I., Kirchhof B., Liakopoulos S., Fauser S. (2014). Nongenetic risk factors for neovascular age-related macular degeneration. Investig. Ophthalmol. Vis. Sci..

[5] Velilla S., García-Medina J.J., García-Layana A., Dolz-Marco R., Pons-Vázquez S., Pinazo-Durán M.D., Gómez-Ulla F., Arévalo J.F., Díaz-Llopis M., Gallego-Pinazo R. (2013). Smoking and age-related macular degeneration: Review and update. J. Ophthalmol..

[6] Hyman L., Schachat A.P., He Q., Leske M.C., Age-Related Macular Degeneration Risk Factors Study Group (2000). Hypertension, cardiovascular disease, and age-related macular degeneration. Arch. Ophthalmol..

[7] Nagata J., Shiose S., Ishikawa K., Fukui T., Kano K., Mori K., Nakama T., Notomi S., Sonoda K.H. (2023). Clinical Characteristics of Eyes with Neovascular Age-Related Macular Degeneration and Retinal Pigment Epithelium Tears. J. Clin. Med..

[8] Jaffe G.J., Martin D.F., Toth C.A., Daniel E., Maguire M.G., Ying G.S., Grunwald J.E., Huang J. (2013). Macular morphology and visual acuity in the comparison of age-related macular degeneration treatments trials. Ophthalmology.

[9] Sharma S., Toth C.A., Daniel E., Grunwald J.E., Maguire M.G., Ying G.S., Huang J., Martin D.F., Jaffe G.J. (2016). Macular Morphology and Visual Acuity in the Second Year of the Comparison of Age-Related Macular Degeneration Treatments Trials. Ophthalmology.

[10] Sarraf D., London N.J., Khurana R.N., Dugel P.U., Gune S., Hill L., Tuomi L. (2016). Ranibizumab Treatment for Pigment Epithelial Detachment Secondary to Neovascular Age-Related Macular Degeneration: Post Hoc Analysis of the HARBOR Study. Ophthalmology.

[11] Ritter M., Simader C., Bolz M., Deák G.G., Mayr-Sponer U., Sayegh R., Kundi M., Schmidt-Erfurth U.M. (2014). Intraretinal cysts are the most relevant prognostic biomarker in neovascular age-related macular degeneration independent of the therapeutic strategy. Br. J. Ophthalmol..

[12] Simader C., Ritter M., Bolz M., Deák G.G., Mayr-Sponer U., Golbaz I., Kundi M., Schmidt-Erfurth U.M. (2014). Morphologic parameters relevant for visual outcome during anti-angiogenic therapy of neovascular age-related macular degeneration. Ophthalmology.

[13] Waldstein S.M., Wright J., Warburton J., Margaron P., Simader C., Schmidt-Erfurth U. (2016). Predictive Value of Retinal Morphology for Visual Acuity Outcomes of Different Ranibizumab Treatment Regimens for Neovascular AMD. Ophthalmology.

[14] Schmidt-Erfurth U., Waldstein S.M., Deak G.G., Kundi M., Simader C. (2015). Pigment epithelial detachment followed by retinal cystoid degeneration leads to vision loss in treatment of neovascular age-related macular degeneration. Ophthalmology.

[15] Waldstein S.M., Simader C., Staurenghi G., Chong N.V., Mitchell P., Jaffe G.J., Lu C., Katz T.A., Schmidt-Erfurth U. (2016). Morphology and Visual Acuity in Aflibercept and Ranibizumab Therapy for Neovascular Age-Related Macular Degeneration in the VIEW Trials. Ophthalmology.

[16] Ashraf M., Souka A., Adelman R.A. (2018). Age-related macular degeneration: Using morphological predictors to modify current treatment protocols. Acta Ophthalmol..

[17] Khanani A.M., Eichenbaum D., Schlottmann P.G., Tuomi L., Sarraf D. (2018). Optimal management of pigment epithelial detachments in eyes with neovascular age-related macular degeneration. Retina.

[18] Karampelas M., Malamos P., Petrou P., Georgalas I., Papaconstantinou D., Brouzas D. (2020). Retinal Pigment Epithelial Detachment in Age-Related Macular Degeneration. Ophthalmol. Ther..

[19] Selvam A., Singh S.R., Arora S., Patel M., Kuchhal A., Shah S., Ong J., Rasheed M.A., Manne S.R., Ibrahim M.N. (2023). Pigment epithelial detachment composition indices (PEDCI) in neovascular age-related macular degeneration. Sci. Rep..

[20] Yannuzzi L.A., Hope-Ross M., Slakter J.S., Guyer D.R., Sorenson J.A., Ho A.C., Sperber D.E., Freund K.B., Orlock D.A. (1994). Analysis of vascularized pigment epithelial detachments using indocyanine green videoangiography. Retina.

[21] The Age-Related Eye Disease Study Research Group (2001). The age-related eye disease study system for classifying age-related macular degeneration from stereoscopic color fundus photographs: The age-related eye disease study report number 6. Am. J. Ophthalmol..

[22] Pepple K., Mruthyunjaya P. (2011). Retinal pigment epithelial detachments in age-related macular degeneration: Classification and therapeutic options. Semin. Ophthalmol..

[23] Pauleikhoff D., Löffert D., Spital G., Radermacher M., Dohrmann J., Lommatzsch A., Bird A.C. (2002). Pigment epithelial detachment in the elderly. Clinical differentiation, natural course and pathogenetic implications. Graefes Arch. Clin. Exp. Ophthalmol..

[24] Shu Y., Ye F., Liu H., Wei J., Sun X. (2023). Predictive value of pigment epithelial detachment markers for visual acuity outcomes in neovascular age-related macular degeneration. BMC Ophthalmol..

[25] Macular Photocoagulation Study Group (1982). Argon laser photocoagulation for senile macular degeneration. Results of a randomized clinical trial. Arch. Ophthalmol..

[26] Donati G., Kapetanios A.D., Pournaras C.J. (1999). Principles of treatment of choroidal neovascularization with photodynamic therapy in age-related macular degeneration. Semin. Ophthalmol..

[27] Stanga P.E., Valentín-Bravo F.J., Stanga S.E.F., Reinstein U.I., Pastor-Idoate S., Downes S.M. (2023). Faricimab in neovascular AMD: First report of real-world outcomes in an independent retina clinic. Eye.

[28] Yeung L., Hsieh Y.T., Yang C.H., Chen L.J., Chen S.J., Cheng C.K., Sheu S.J., Tsai C.Y., Wu T.T., Wu W.C. (2021). Management of neovascular age-related macular degeneration: Taiwan expert consensus. J. Formos. Med. Assoc..

[29] Schmidt-Erfurth U., Chong V., Loewenstein A., Larsen M., Souied E., Schlingemann R., Eldem B., Monés J., Richard G., Bandello F. (2014). Guidelines for the management of neovascular age-related macular degeneration by the European Society of Retina Specialists (EURETINA). Br. J. Ophthalmol..

[30] Flaxel C.J., Adelman R.A., Bailey S.T., Fawzi A., Lim J.I., Vemulakonda G.A., Ying G.S. (2020). Age-Related Macular Degeneration Preferred Practice Pattern^®^. Ophthalmology.

[31] Clearkin L., Ramasamy B., Wason J., Tiew S. (2019). Anti-VEGF intervention in neovascular AMD: Benefits and risks restated as natural frequencies. BMJ Open Ophthalmol..

[32] Pugazhendhi A., Hubbell M., Jairam P., Ambati B. (2021). Neovascular Macular Degeneration: A Review of Etiology, Risk Factors, and Recent Advances in Research and Therapy. Int. J. Mol. Sci..

[33] William A., Verma-Fuehring R., Kuehnel S., Schwabe D., Kampik D., Goebel W., Hillenkamp J. (2023). Morphological Macular Changes Under Brolucizumab Treatment for Neovascular Age-Related Macular Degeneration Refractory to Previous Anti-VEGF Treatment Compared with Treatment-Naive Eyes. Clin. Ophthalmol..

[34] Fung A.E., Lalwani G.A., Rosenfeld P.J., Dubovy S.R., Michels S., Feuer W.J., Puliafito C.A., Davis J.L., Flynn H.W., Esquiabro M. (2007). An optical coherence tomography-guided, variable dosing regimen with intravitreal ranibizumab (Lucentis) for neovascular age-related macular degeneration. Am. J. Ophthalmol..

[35] Akkan Aydoğmuş F.S., Onwuka O., Saddemi J., Lasalle C.C., Ramsey D.J. (2023). Second eyes to develop neovascular age-related macular degeneration have fewer symptoms and better one-year visual outcomes. BMC Ophthalmol..

[36] Cheong K.X., Teo K.Y.C., Cheung C.M.G. (2021). Influence of pigment epithelial detachment on visual acuity in neovascular age-related macular degeneration. Surv. Ophthalmol..

[37] Sun Z., Yang Y., Lin B., Huang Y., Zhou R., Yang C., Li Y., Huang S., Liu X. (2023). Comparative efficacy of aflibercept and ranibizumab in the treatment of age-related macular degeneration with retinal pigment epithelial detachment: A systematic review and network meta-analysis. BMC Ophthalmol..

[38] Karampelas M., Syriga M., Petrou P., Georgalas I., Papaconstantinou D., Brouzas D. (2022). Morphometric analysis of fibrovascular pigment epithelial detachments treated with ranibizumab and aflibercept. Eur. J. Ophthalmol..

[39] Hamid M.A., Abdelfattah N.S., Salamzadeh J., Abdelaziz S.T.A., Sabry A.M., Mourad K.M., Shehab A.A., Kuppermann B.D. (2021). Aflibercept therapy for exudative age-related macular degeneration resistant to bevacizumab and ranibizumab. Int. J. Retin. Vitr..

[40] Zhou H., Zhao X., Wang S., Chen Y. (2023). Determination of Vascular Endothelial Growth Factor-B Concentrations in Aqueous Humor and Plasma of Neovascular Age-Related Macular Degeneration and Polypoidal Choroidal Vasculopathy Patients Before and After Anti-VEGF Therapy. Ophthalmol. Ther..

[41] Wykoff C.C., Garmo V., Tabano D., Menezes A., Kim E., Fevrier H.B., LaPrise A., Leng T. (2024). Impact of Anti-VEGF Treatment and Patient Characteristics on Vision Outcomes in Neovascular Age-related Macular Degeneration: Up to 6-Year Analysis of the AAO IRIS^®^ Registry. Ophthalmol. Sci..

[42] Lai T.T., Hsieh Y.T., Yang C.M., Ho T.C., Yang C.H. (2019). Biomarkers of optical coherence tomography in evaluating the treatment outcomes of neovascular age-related macular degeneration: A real-world study. Sci. Rep..

[43] Cho H.J., Kim K.M., Kim H.S., Lee D.W., Kim C.G., Kim J.W. (2016). Response of Pigment Epithelial Detachment to Anti-Vascular Endothelial Growth Factor Treatment in Age-Related Macular Degeneration. Am. J. Ophthalmol..

[44] Inan S., Polat O., Karadas M., Inan U.U. (2019). The association of exudation pattern with anatomical and functional outcomes in patients with Neovascular Age-Related Macular Degeneration. Rom. J. Ophthalmol..

[45] Leung K.F.C., Downes S.M., Chong V. (2018). A Retrospective Analysis of the Effect of Subretinal Hyper-Reflective Material and Other Morphological Features of Neovascular Age-Related Macular Degeneration on Visual Acuity Outcomes in Eyes Treated with Intravitreal Aflibercept over One Year. Vision.

[46] Tarakcioglu H.N., Ozkaya A., Kemer B., Taskapili M. (2019). Multimodal imaging based biomarkers predictive of early and late response to anti-VEGFs during the first year of treatment for neovascular age-related macular degeneration. J. Fr. Ophtalmol..

[47] Dervenis N., Younis S. (2016). Macular morphology and response to ranibizumab treatment in patients with wet age-related macular degeneration. Clin. Ophthalmol..

[48] Mathew R., Richardson M., Sivaprasad S. (2013). Predictive value of spectral-domain optical coherence tomography features in assessment of visual prognosis in eyes with neovascular age-related macular degeneration treated with ranibizumab. Am. J. Ophthalmol..

[49] Blanco-Garavito R., Jung C., Uzzan J., Quaranta-ElMaftouhi M., Coscas F., Sahel J., Korobelnik J.F., Béchet S., Querques G., Souied E.H. (2018). Aflibercept after ranibizumab intravitreal injections in exudative age-related macular degeneration: The ARI2 Study. Retina.

[50] Chen X., Al-Sheikh M., Chan C.K., Hariri A.H., Abraham P., Lalezary M., Lin S.G., Sadda S., Sarraf D. (2016). Type 1 versus type 3 neovascularization in pigment epithelial detachments associated with age-related macular degeneration after anti-vascular endothelial growth factor therapy: A Prospective Study. Retina.

[51] Veritti D., Sarao V., Parravano M., Arias L., Varano M., Lanzetta P. (2017). One-year results of aflibercept in vascularized pigment epithelium detachment due to neovascular AMD: A prospective study. Eur. J. Ophthalmol..

[52] Balaskas K., Karampelas M., Horani M., Hotu O., Keane P., Aslam T. (2017). Quantitative analysis of pigment epithelial detachment response to different anti-vascular endothelial growth factor agents in wet age-related macular degeneration. Retina.

[53] Dirani A., Ambresin A., Marchionno L., Decugis D., Mantel I. (2015). Factors Influencing the Treatment Response of Pigment Epithelium Detachment in Age-Related Macular Degeneration. Am. J. Ophthalmol..

[54] Freeman W.R., Kozak I., Yuson R.M., Nigam N., Cheng L., Mojana F. (2011). Prognosti implications of pigment epithelial detachment in bevacizumab (avastin)-treated eyes with age-related macular degeneration and choroidal neovascularization. Retina.

[55] Arora S., McKibbin M. (2011). One-year outcome after intravitreal ranibizumab for large, serous pigment epithelial detachment secondary to age-related macular degeneration. Eye.

[56] Ach T., Hoeh A.E., Ruppenstein M., Kretz F.T., Dithmar S. (2010). Intravitreal bevacizumab in vascular pigment epithelium detachment as a result of subfoveal occult choroidal neovascularization in age-related macular degeneration. Retina.

[57] Cheong K.X., Grewal D.S., Teo K.Y.C., Gan A.T.L., Jaffe G.J., Cheung G.C.M. (2020). The relationship between pigment epithelial detachment and visual outcome in neovascular age-related macular degeneration and polypoidal choroidal vasculopathy. Eye.

[58] Chan C.K., Sarraf D., Abraham P. (2018). Treatment outcomes of conventional or high-dose ranibizumab for vascularized pigment epithelial detachment based on lesion subtypes. Eur. J. Ophthalmol..

[59] Or C., Chui L., Fallah N., Forooghian F. (2016). Volumetric assessment of the responsiveness of pigment epithelial detachments in neovascular age-related macular degeneration to intravitreal bevacizumab. Retina.

[60] Inoue M., Arakawa A., Yamane S., Kadonosono K. (2013). Variable response of vascularized pigment epithelial detachments to ranibizumab based on lesion subtypes, including polypoidal choroidal vasculopathy. Retina.

[61] Mukai R., Honjo J., Tanaka K., Sekiryu T. (2024). Exploring the comparative regressive effects of aflibercept and faricimab on pigment epithelial detachment. BMC Ophthalmol..

